# Effects of Gestational Magnetic Resonance Imaging on Methylation Status of Leptin Promoter in the Placenta and Cord Blood

**DOI:** 10.1371/journal.pone.0147371

**Published:** 2016-01-20

**Authors:** Ying Wang, Feng-Shan Yan, Jian-Min Lian, She-Wei Dou

**Affiliations:** Department of Radiology, People's Hospital of Zhengzhou University, Henan Provincial People’s Hospital, Zhengzhou, Henan, PR. China; University of Florida, UNITED STATES

## Abstract

Over the past two decades, magnetic resonance imaging (MRI) has been widely used for diagnosis in gestational women. Though it has several advantages, animal and human studies on the safety of MRI for the fetus remain inconclusive. Epigenetic modifications, which are crucial for cellular functioning, are prone to being affected by environmental changes. Therefore, we hypothesized that MRI during gestation may cause epigenetic modification alterations. Here, we investigated DNA methylation patterns of leptin promoter in the placenta and cord blood of women exposed to MRI during gestation. Results showed that average methylation levels of leptin in the placenta and cord blood were not affected by MRI. We also found that the methylation levels in the placenta and cord blood were not affected by different magnetic fields (1.5T and 3.0T MRI). However, if pregnant women were exposed to MRI at 15 to 20 weeks of gestation, the methylation level of leptin in cord blood was visibly lower than that of pregnant women exposed to MRI after 20-weeks of gestation (P = 0.037). mRNA expression level of leptin in cord blood was also altered, though mRNA expression of leptin in the placenta was not significantly affected. Therefore, we concluded that gestational MRI may not have major effects on the methylation level of leptin in cord blood and the placenta except for MRI applied before 20 weeks of gestation.

## Introduction

Since its first successful application in the 1980s, fetal magnetic resonance imaging (MRI) has become a crucial adjuvant- imaging tool in the evaluation of fetal development, which involves imaging the moving fetus without sedation [1, 2]. However, there is no direct evidence to confirm that fetal MRI does not produce harmful effects on the fetus. The long-term safety of fetal MRI has not yet been demonstrated. To date, there is no consensus about the risk of MRI to the fetus. A previous study showed that crown-rump length was affected in mid-gestational mice exposed to 0.35T MRI [3]. Another study showed no effect on embryo mortality, hatching rate or vitality in chickens when the chick embryos were exposed to MRI at different static and time-varying gradient field strengths [4]. However, another study showed that abnormality and mortality rate increased in 6-day chick embryos exposed to 1.5T MRI [5]. Similar contradictory results were reported by studies on animals [6, 7]. In humans, previous studies have indicated that fetal growth and the health of the child are not influenced by exposure to MRI during gestation [8, 9]. Several studies have monitored the variability in fetal heart rate after pregnant mothers were exposed to MRI, but no effects were reproted [10–12].

Barker suggests that diseases in adulthood, especially metabolic diseases, originate at the early gestational stage [13]. Epigenetic factors, including DNA methylation, histone modification, and non-coding RNA, may be affected by MRI during gestation because epigenetic modifications are prone to be affected by external and internal factors [14–16]. Epimutation caused by abnormal intrauterine environment may play a key role in the long-term effects on health during adulthood [17–19].

Leptin is involved in food intake, energy expenditure, and reproduction amongst many other functions, and is associated with several metabolic diseases, such as obesity [20, 21]. In adipose tissue of diet-induced obese mice, leptin promoter methylation at CpG sites increases and the changed level of leptin expression is closely associated with altered epigenetic modification in leptin promoter [22]. In humans, the concentration of circulating leptin is altered by obesity and the promoter methylation of leptin is also altered in obese individuals [23].

Leptin is mainly produced by adipose tissue. During pregnancy, leptin in serum is produced by fetal and maternal adipose tissue and placenta [24–26]. If methylation levels in fetal promoter of leptin is changed during pregnancy, fetal development may be affected and the methylation status in the leptin promoter may also be altered in fetal tissues. Previous studies have shown that the fetal methylation level of leptin could be affected by the intrauterine environment [27, 28]. Gestational high-fat-diet decreases promoter methylation of leptin in the offspring [29].Therefore, we hypothesize that MRI may affect the DNA methylation of leptin in cord blood and the placenta during gestation. In this study, we investigated the methylation level of the leptin promoter in the cord blood and the placenta in response to exposure to MRI during gestation.

## Materials and Methods

### Subjects and MRI examination

This study was reviewed and approved by the Life Science Ethics Committee of Zhengzhou University. Written informed consents were obtained from all subjects. 55 pregnant women, who underwent MRI (exposed to MRI group, [EG]) for maternal or fetal reasons between March 2011 and November 2014, were enrolled in this study. Additionally, 62 women who were not exposed to MRI during gestation were enrolled as the control group (non-exposed to MRI group, [NEG]), matched with characteristics as those in the EG group. Characteristics of the pregnancies are shown in Table 1. Subjects were selected based on the following criteria: singleton pregnancy, no gestational diseases (*e*.*g*. gestational diabetes, hypertension.), maternal age > 18 years, non-smoking, no life-threatening complications in the mother, and no congenital or chromosomal abnormalities in the infant. Clinical information including personal and family medical history, lifestyle, exposure history, pre-pregnancy body mass index (BMI), and tobacco use, was collected using a structured chart review form and an interviewer-administered questionnaire.

**Table 1:** Characteristics of subjects.

	Variable	EG		NEG
		EE	LE	
	**No.of subjects**	17	38	62
	**Average age (year)**	29.32±1.89	28.06±2.97	30.23±1.97
	**Average BMI**	21.21±2.44	21.85±3.11	22.37±3.04
	**Average gestational age (wk) exposed to MRI**	19.2±1.83	29.9±2.17	-
	**gestational disease**	no	no	-
	**Head**	0	5	-
	**Spine**	3	6	-
	**Waist**	2	6	-
**No. of MRI position**	**Fetus**	4	7	-
	**Chest**	1	5	-
	**Abdomen**	4	1	-
	**Pelvimetry**	3	7	-
	**Shoulder**	0	1	-
**No. of Magnetic field**	**1.5T**	10	23	-
	**3.0T**	7	15	-
**No. of examination per pregnancy**	**One**	11	25	-
	**Two or more**	6	13	-
	**Average examination time (min)**	11.8±3.42	13.2±2.77	-
	**Average birth weight**	2.43±0.81	3.01±0.65	2.97±0.78
	**Average gestational age (wk)**	37.7±1.23	38.1±1.44	38.0±1.58

Note: NEG, nonexposed to MRI group; EG, exposed to MRI group; EE, exposed to MRI at 15-20wk of gestation; LE, exposed to MRI after 20wk of gestation.

MRI was performed using a clinical 1.5T or 3.0T machine (Siemens, Germany) according to the doctor’s advice. Subjects were scanned in the supine position. For fetal imaging, the protocol was determined according to the indication for the examination.

### Sample collection

Fetal placenta samples were collected from all subjects within two hours after delivery. Placenta samples were placed immediately in RNAlater solution (Sigma-Aldrich, China), and stored at 4°C. The placental tissues were then blotted dry, frozen in liquid nitrogen, and stored at -80°C until use. Cord blood was collected from the umbilical cord and stored at -80°C until use.

### DNA purification and bisulfite modification

DNA from placental tissues and cord blood was extracted using DNAeasy Blood & Tissue Kit (Tiangen, China) according to the manufacturer protocol. The purified DNA was stored at -20°C until use. DNA samples were modified using the EZ DNA methylation Kit (Zymo Research, USA) according to the manufacturer protocol.

### Pyrosequencing and bisulfite sequencing (BS)

Methylation level was evaluated by pyrosequencing. Primers (Invitrogen, China) were designed using the PyroMark Assay Design software version 2.0.1.15 (Qiagen) for the region that regulates the expression of leptin [30, 31]. The target fragment length was 383bp and 23 CpG sites were analyzed. The Primer [26] details are shown in Table 2. Pyrosequencing was performed using the Pyromark MD (Qiagen) instrument (BGI Tech, China). Primers for BS were designed using Methprimer (http://www.urogene.org/cgi-bin/methprimer/methprimer.cgi) and described in Table 2. Bisulfite DNA was amplified with BS primers and sequenced (Invitrogen, China). The samples from subjects exposed to MRI after 20 weeks of gestation (LE group, n = 38) and those exposed to MRI at 15–20 weeks (EE group, n = 17) of gestation were prepared. Each individual sample was treated using methylation kit and amplified by nested polymerase chain reaction (-PCR). The products from EE and LE samples were pooled together, respectively. The pooled products were cloned in T-vector. Cloning was repeated three times, separately. Then, monoclones were selected and sequenced. At least ten clones were sequenced for each pool.

**Table 2:** Primers sequences for pyrosequencing and bisulfite sequencing (BS).

Primer	Sequence
**PCR Forward**	5'- GAGTTTTTGGAGGGATATTAAGGAT-3'
**PCR Reverse**	5'- CAAAATTATATAAAACCCTATAACCTACCA-3'
**Pyrosequencing 1**	5'-GGGAGGTATTTAAGGG-3'
**Pyrosequencing 2**	5'-GGGAGGGGAGGGAGTTGG-3'
**BS Forward**	5'-TTTTTTAATTTTGGGTTTTTTTGG-3'
**BS Reverse**	5'-CAAAATTATATAAAACCCTATAACCTACCA-3'

### Purification of total RNA and quantitative real-time PCR (qRT-PCR)

Total RNA from placenta and cord blood was extracted using RNeasy Plus Micro Kit (Qiagen). First strand cDNA was synthesized using RT First Strand Kit (Qiagen), which was then used as template to test the expression of leptin. Relative expression of leptin was checked using Applied Biosystems® 7500 Real-Time PCR Systems (ABI) and β-actin was used as control. Primers used are as follows: leptin, 5’-TGCCTTCCAGAAACGTGATCC-3’,5’-CTCTGTGGAGTAGCCTGAAGC-3’;β-actin, 5'-CTACAATGAGCTGCGTGTGG-3',5'-TAGCTCTTCTCCAGGGAGGA-3'. mRNA expression was calculated by 2^-⊿⊿Ct^.

### Statistical analysis

Average data, such as age, BMI, gestational age, expression of leptin and methylation level of pyrosequencing, were presented as mean ± SD. The difference was examined by independent sample two-tail t-test. Results of BS were presented as percentage values and the difference was evaluated by Chi-square test. The statistic difference of the expression of leptin was tested using One-Way ANOVA. P < 0.05 was considered as statistically significant.

## Results

### Characteristics of subjects

The average age between EG (n = 55) and NEG (n = 62) was similar (28.06 ± 2.97 & 30.23 ± 1.97, P = 0.382). The pre-pregnancy BMI ranged from 17.7 to 25.2, the average BMI between the two groups was 21.21 ± 2.44 and 22.37 ± 3.04 (P = 0.457).The average gestational age between EG and NEG was similar (P = 0.346). Gestational diseases, such as gestational diabetes and hypertension, were not diagnosed. None of the enrolled objects had a history of smoking. No subjects were reported to have any diseases that could affect epigenetic modifications pre- and post-gestation. The relative characteristics are presented in Table 1.

### Average methylation level of leptin is not affected by MRI

**Fig 1:**
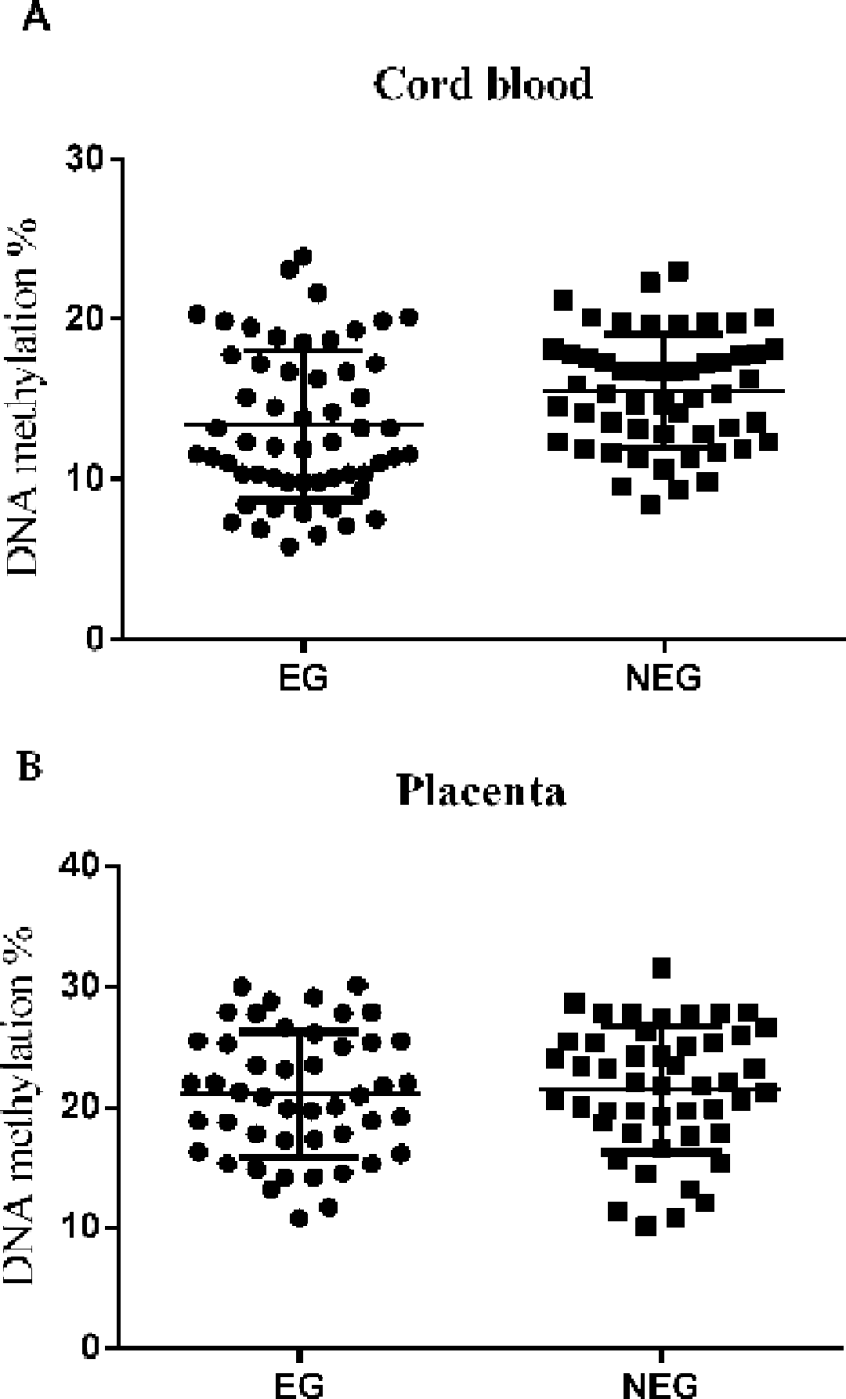
Methylation levels of the leptin promoter in cord blood and the placenta (A): Average methylation level of leptin in cord blood from EG and NEG; (B): Average methylation level in placenta of EG and NEG. EG, exposed to MRI during gestation; NEG, not exposed to MRI during gestation.

For EG group, the average methylation level of leptin in cord blood (13.35 ± 4.72%) was similar to that for the NEG group (15.46 ± 3.67%, P = 0.509, Fig 1A). In the placenta, the average methylation level in leptin promoter was 22.3 ± 4.18% and 23.7 ± 3.58% for EG and NEG, respectively (Fig 1B, P = 0.419).

### Average methylation level is similar between pregnancies exposed to 1.5T and 3.0T

**Fig 2:**
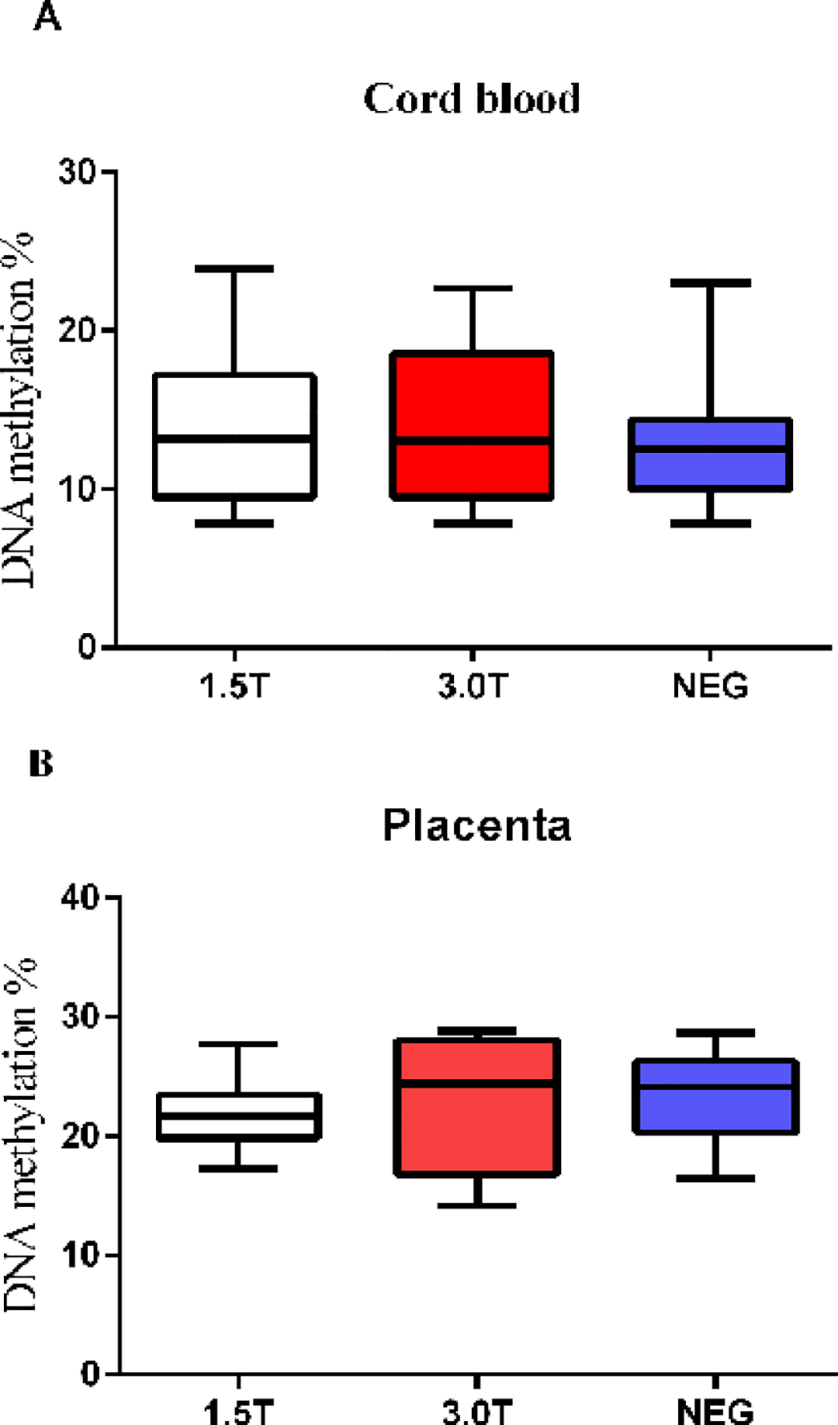
Methylation levels in cord blood and the placenta of pregnant women exposed to MRI. (A): Average methylation level of leptin in cord blood of pregnancies exposed to 1.5T MRI; (B): Mean methylation level in placenta of gestational women exposed to 3.0T MRI.

We further compared the average methylation levels in cord blood and the placenta of women exposed to 1.5T (n = 33) and 3.0T (n = 22) MRI during gestation. Results showed that the mean methylation in cord blood was 13.8 ± 4.73% and 14.0 ± 5.59% for 1.5T and 3.0T MRI (P = 0.944, Fig 2A), respectively. In the placenta, the mean methylation level was 21.8 ± 2.97% for 1.5T MRI and 23.1 ± 6.06% for 3.0T MRI (P = 0.634, Fig 2B).

### Early pregnancy exposure to MRI has adverse effects on methylation of leptin in cord blood

**Fig 3:**
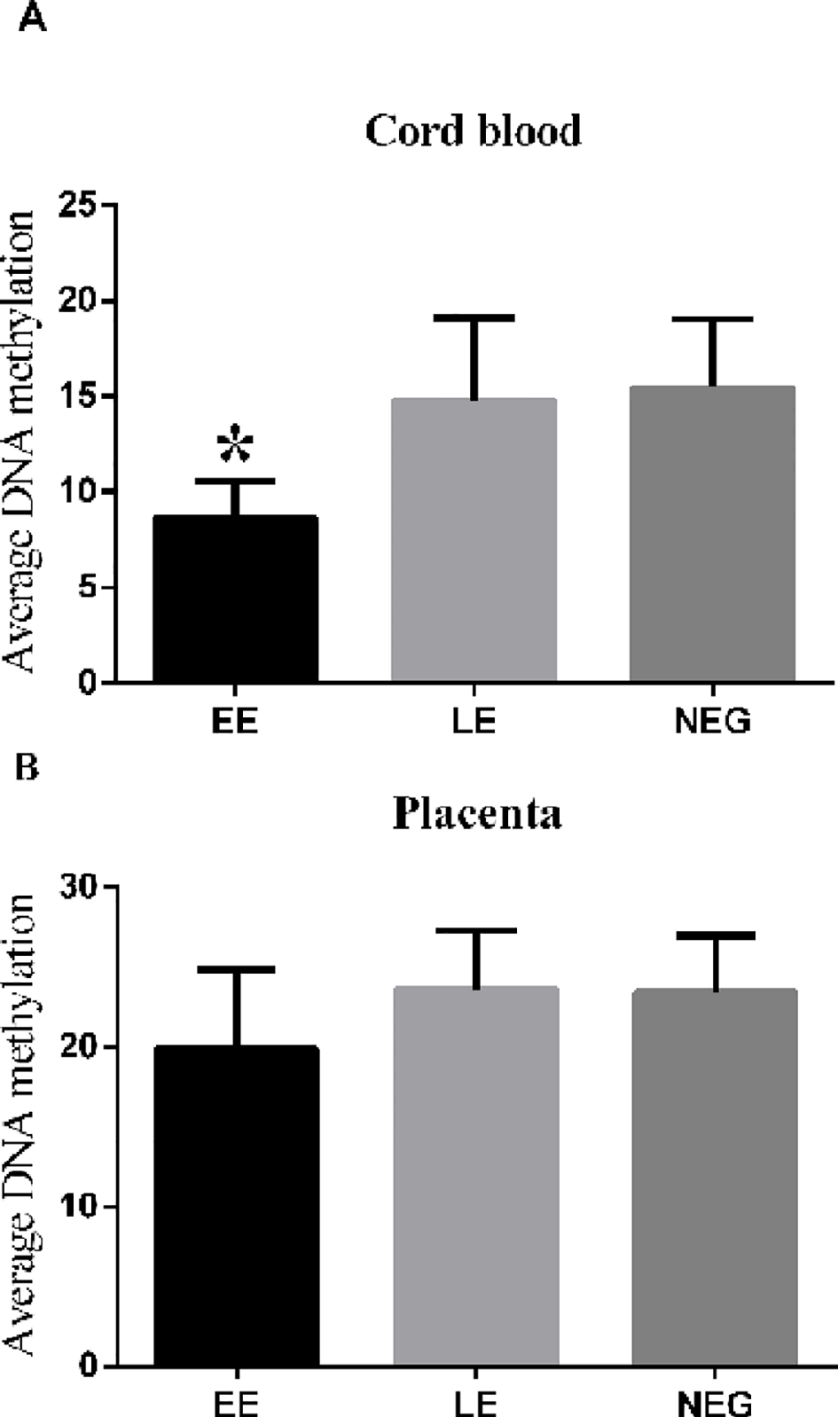
Average methylation level in cord blood and the placenta from EE and LE. (A): Average methylation level of leptin in cord blood from EE, NEG and LE; (B): Mean methylation level in placenta of EE, NEG and LE. EE, early pregnancy exposed to MRI (15–20 weeks of gestation); LE, late pregnancy exposed to MRI (after 20 weeks of gestation); NEG, non-exposed to MRI during gestation; * P < 0.05.

We found that the mean methylation level in cord blood (8.7 ± 3.47%) of EE (n = 17) was significantly lower than that in NEG (n = 62) and LE (15.5 ± 3.67% versus 14.8 ± 4.30%, P = 0.037, n = 38, Fig 3A). The methylation level in placenta in EE (19.9 ± 4.99%) was similar to that in NEG and LE (23.3 ± 3.58% versus 21.4 ± 6.45% respectively, P = 0.706, Fig 3B).

**Fig 4:**
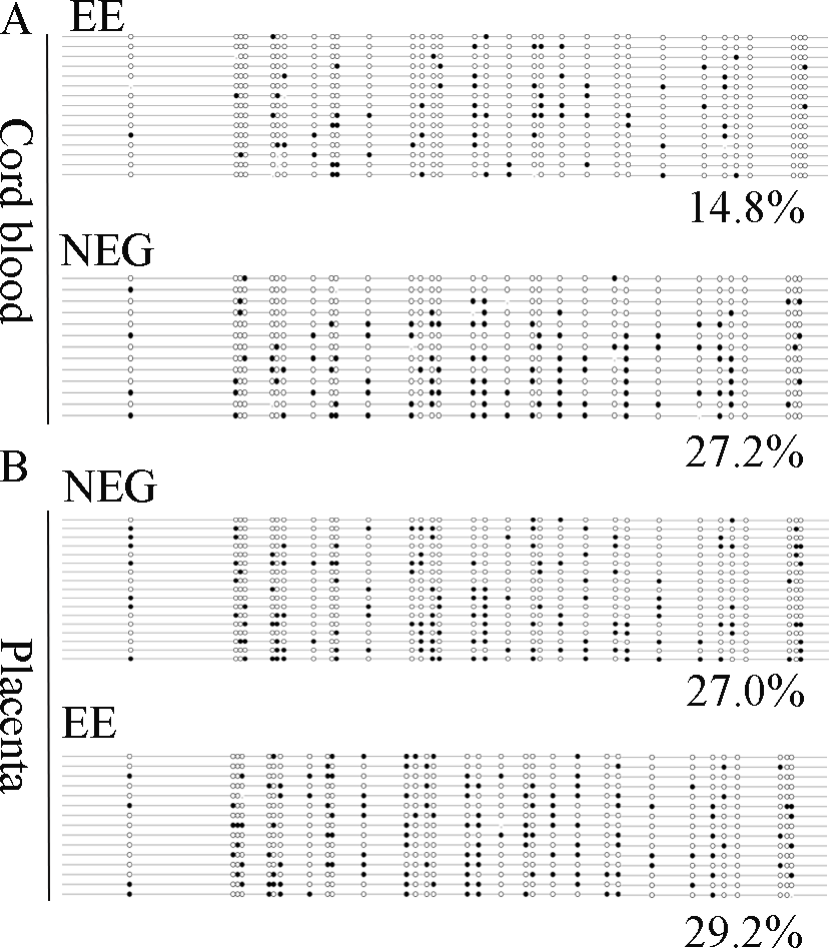
Methylation patterns of leptin promoter in cord blood and placenta. The methylation status of leptin in cord blood and placenta of EE and NEG were further analyzed by BS. (A): Methylation patterns of leptin in cord blood of EE and NEG; (B): Methylation patterns of leptin in placenta of EE and NEG. EE, early pregnancy exposed to MRI (15–20 weeks of gestation); NEG, non-exposed to MRI during gestation; number, indicating the average methylation of leptin; white circle, unmethylated CpG site; black circle, methylated CpG site.

To further confirm this result, we examined the methylation level in cord blood and the placenta by BS. As shown in Fig 4A, EE presented lower methylation levels in cord blood (14.8%, P < 0.001) as compared to NEG (27.2%). In placenta, methylation level was similar between EE and NEG (27.0% versus 29.2% respectively, P = 0.409, Fig 4B).

We also analyzed the birth weight of babies. We found that the average birth weight was not influenced by exposure to MRI during gestation (P = 0.19), although it was slightly lower in EG than that in NEG. The average birth weight of EE was also slightly lower than that of LE (P = 0.28, Table 1).

**Fig 5:**
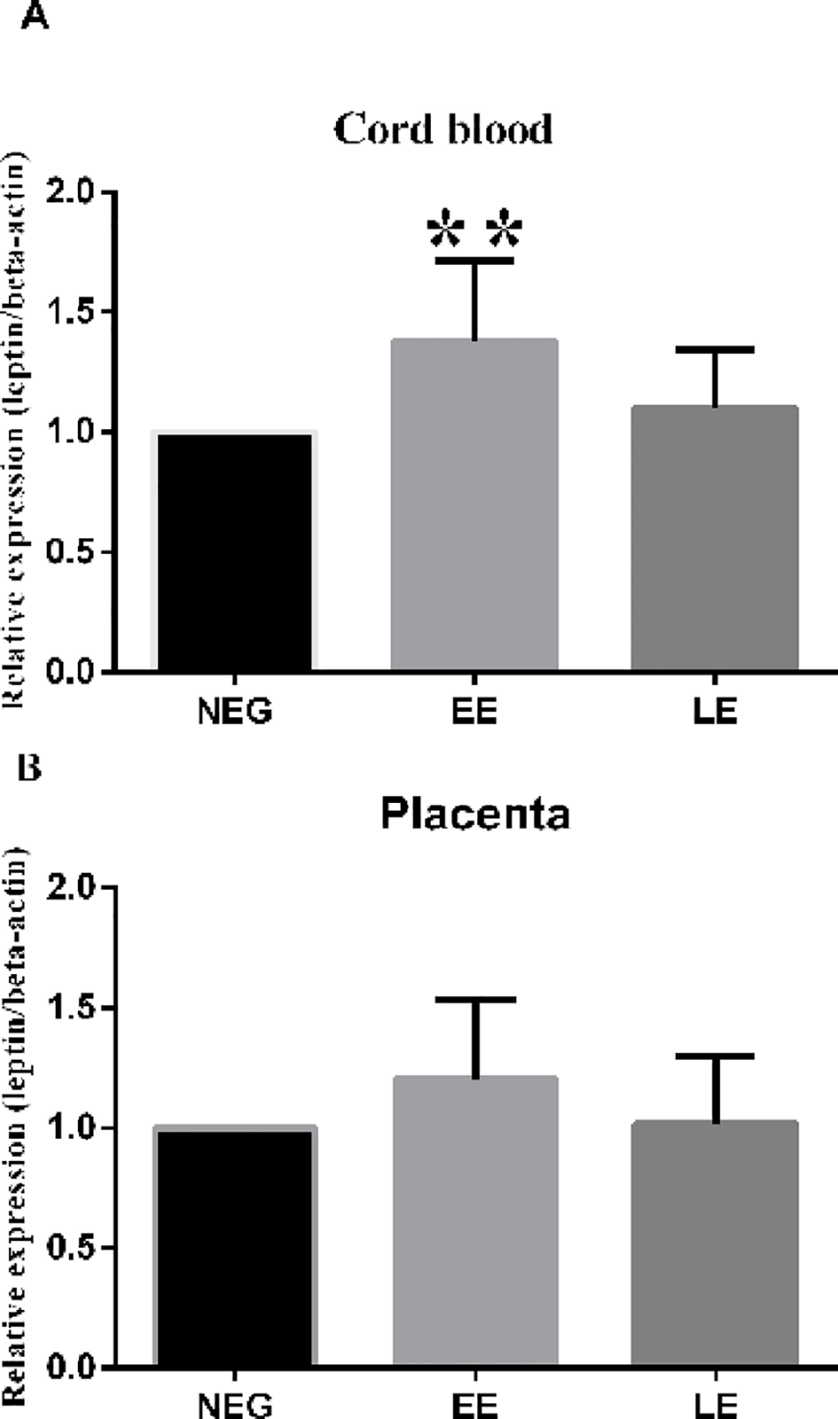
Relative expression of leptin in cord blood and the placenta. Expression of leptin mRNA in cord blood and the placenta was tested by qRT-PCR. (A), leptin mRNA expression in cord blood; (B), leptin mRNA expression in the placenta. EE, early pregnancy exposed to MRI (15–20 weeks of gestation); LE, late pregnancy exposed to MRI (after 20 weeks of gestation); NEG, non-exposed to MRI during gestation; ** P < 0.01.

We further checked the mRNA level of leptin in cord blood and the placenta. In the cord blood, mRNA level of leptin in EE (1.38±0.30) was higher than that in LE (1.10±0.24, P = 0.007, Fig 5A). In the placenta, mRNA level of leptin in EE (1.20±0.33) was slightly higher that in LE (1.02±0.28, P = 0.08, Fig 5B). The expression of LE in cord blood and the placenta was similar to that of NEG (P = 0.47 and 0.98).

## Discussion

The viewpoint on fetal safety of MRI-exposed pregnant women is still not consistent because results from animals are contradictory to that from humans. Furthermore, the sample sizes in previous studies were limited and the effects of variation of MRI field strengths were not perfectly evaluated. In the present study, we examined the methylation level of the leptin promoter in cord blood and the placenta of women exposed to MRI during gestation. We found that the average methylation of leptin in cord blood and the placenta was not affected by MRI after 20 weeks of gestation. We also found that the methylation level of leptin in cord blood and the placenta was similar between women who were exposed to 1.5T or 3.0T MRI. These results agree with those of previous studies. For example, Barker et al. [8] performed a three-year follow up study of children imaged *in utero*; evidence showed that there was no increase in disease or disability in them [8]. Other studies also demonstrated that intrauterine fetal growth was not influenced by MRI during gestation [9, 10].

Triulzi et al. reviewed the indications of fetal MRI and suggested that fetal MRI is not recommended prior to 19 weeks of gestation [32]. The United States’ Food and Drug Administration (FDA), the International Commission on Nonionizing Radiation Protection and the Medical Device Agency of the United Kingdom suggest that fetal MRI should not be performed during early pregnancy because it may have deleterious effects on embryonic development [33–36]. However, MRI cannot be completely avoided for women during early pregnancy. In the present study, we analyzed the methylation level of the leptin promoter in cord blood and the placenta of women exposed to MRI at 15 to 20 weeks of gestation. We found that the methylation level in promoter of leptin in cord blood of EE was significantly lower than that in LE, but there was no obvious change in promoter methylation of placental leptin because the effects of MRI on different tissues may be different.

Leptin is predominantly secreted by adipose tissue. During pregnancy, the placenta can also produce leptin [25]. Leptin is not only crucial for adults, but also for fetal development during gestation [37]. Studies have indicated that serum leptin concentration in offspring is prone to be affected by maternal life and nutrients during pregnancy [38, 39]. Additionally, this altered serum leptin concentration is associated with the methylation status in leptin promoter since leptin expression is regulated by DNA methylation levels in the promoter. A survey of the effects of MRI on pregnancies showed that birth weight for pregnancies exposed to MRI during gestation was lower by 2.5kg compared to those not exposed [40]. However, another study showed that the birth weight was not affected by MRI [41]. In this study, we found that the birth weight in EG was not significantly different from that in NEG (P = 0.19). In addition, the birth weight in EE was slightly lower than that in NEG and LE (P = 0.28). Another previous study showed that the mRNA and protein level of leptin was higher in the placenta of smaller twins than that in normal twins [37]. We also found that the mRNA level of leptin in the cord blood from EE was higher than those from LE and NEG, and the expression of leptin in the placenta in EE was slightly higher than that in LE and NEG These results may partly elucidate why the weight of newborns in EE was slightly lower than that in LE. However, only 17 of 55 women were exposed to MRI at 15 to 20 weeks of gestation in this study, because MRI was not suggested, especially for those at an early gestational stage. Although the cord blood, placenta and fetus originate from the same fertilized oocyte, the changed promoter methylation status of leptin in cord blood could not absolutely confirm the promoter methylation of leptin in fetal adipose tissue is also altered by gestational MRI. Therefore, more studies are needed to elucidate the effects of MRI on promoter methylation of leptin in adipose tissue.

A previous study indicates that offspring obesity is associated with abnormal expression of leptin [39]. Although we did not obtain direct data to confirm if the promoter methylation of leptin in fetal tissues was altered by gestational MRI, our data indirectly reflects that the future health of the offspring in EE might be affected. The health of babies in EE was not monitored in this study. Thus, we are unable to conclude whether the long-term health of children might be affected because if the methylation level in fetal promoter of leptin is altered for a few individuls, the long-term effects on health may not be observed in a short time. Therefore, it is crucial to monitor the future health of children exposed to MRI during pregnancy.

In summary, the average methylation in cord blood and the placenta was not significantly affected by MRI during gestation, although the methylation level of leptin was altered at 15 to 20 weeks of gestation. Furthermore, the relevance of our findings and the long-term health of individuls exposed to MRI during early gestation is not clear. Therefore, more studies are needed to present the safety of MRI on fetus exposed to MRI during early gestation.
